# Nucleosides Present on Phlebotomine Saliva Induce Immunossuppression and Promote the Infection Establishment

**DOI:** 10.1371/journal.pntd.0003600

**Published:** 2015-04-07

**Authors:** Vanessa Carregaro, José M. Ribeiro, Jesus G. Valenzuela, Djalma L. Souza-Júnior, Diego L. Costa, Carlo J. F. Oliveira, Laís A. Sacramento, Manuela S. L. Nascimento, Cristiane M. Milanezi, Fernando Q. Cunha, João S. Silva

**Affiliations:** 1 Department of Biochemistry and Immunology, School of Medicine of Ribeirão Preto, University of São Paulo, Ribeirão Preto, Brazil; 2 Section of Vector Biology, Laboratory of Malaria and Vector Research, National Institute of Allergy and Infectious Diseases, National Institutes of Health, Bethesda, Maryland, United States of America; 3 Vector Molecular Biology Unit, Laboratory of Malaria and Vector Research, National Institute of Allergy and Infectious Diseases, National Institutes of Health, Bethesda, Maryland, United States of America; 4 Institute of Biological and Natural Sciences, Federal University of Triângulo Mineiro, Uberaba, Brazil; 5 Department of Pharmacology, School of Medicine of Ribeirão Preto, University of São Paulo, Ribeirão Preto, Brazil; Liverpool School of Tropical Medicine, UNITED KINGDOM

## Abstract

**Background:**

Sand fly saliva plays a crucial role in establishing Leishmania infection. We identified adenosine (ADO) and adenosine monophosphate (AMP) as active pharmacologic compounds present in *Phlebotomus papatasi* saliva that inhibit dendritic cell (DC) functions through a PGE_2_/IL 10-dependent mechanism.

**Methodology/Principal Findings:**

Herein, we prepared a mixture of ADO and AMP in equimolar amounts similar to those present in the salivary-gland extract (SGE) form one pair of salivary glands of *P*. *papatasi* and co-injected it with *Leishmania amazonensis* or *L*. *major* into mouse ears. ADO+AMP mimicked exacerbative effects of *P*. *papatasi* saliva in leishmaniasis, increasing parasite burden and cutaneous lesions. Enzymatic catabolism of salivary nucleosides reversed the SGE-induced immunosuppressive effect associated with IL-10 enhancement. Immunosuppressive factors COX2 and IL-10 were upregulated and failed to enhance ear lesion and parasite burden in IL 10^-/-^ infected mice. Furthermore, nucleosides increased regulatory T cell (Treg) marker expression on CD4^+^CD25^-^ cells, suggesting induction of Tregs on effector T cells (T eff). Treg induction (iTreg) was associated with nucleoside-induced tolerogenic dendritic cells (tDCs) expressing higher levels of COX_2_ and IL-10. In vitro generation of Tregs was more efficient in DCs treated with nucleosides. Suppressive effects of nucleosides during cutaneous leishmaniasis were mediated through an A2_A_R-dependent mechanism. Using BALB/c mice deficient in A2A ADO receptor (A2_A_R^–/–^), we showed that co-inoculated mice controlled infection, displaying lower parasite numbers at infection sites and reduced iTreg generation.

**Conclusion/Significance:**

We have demonstrated that ADO and AMP in *P*. *papatasi* saliva mediate exacerbative effects of *Leishmania* infection by acting preferentially on DCs promoting a tolerogenic profile in DCs and by generating iTregs in inflammatory foci through an A2_A_R mechanism.

## Author Summary

*Leishmania* parasites are transmitted to their vertebrate hosts by infected Phlebotomine sand flies during the blood meal of the flies. During the *Leishmania* transmission, the saliva is inoculated together with parasites and exhibit several pharmacological compounds that facilitate blood feeding, interfering on homeostasis and avoiding inflammation. Thus, these compounds allow the establishment of pathogen infection. We recently identified adenosine (ADO) and adenosine monophosphate (AMP) as major immunomodulatory compounds present within the Old World sand fly species *Phlebotomus papatasii*, which protected mice from extreme inflammatory insults. ADO limits the magnitude of immune response by displaying a potent anti-inflammatory activity. Here, we demonstrated that ADO and AMP present in *Phlebotomus papatasi* saliva are involved in the establishment of parasite infection. Such nucleosides act through adenosine A2_A_ receptor (A2_A_R), inducing a tolerogenic profile on dendritic cells (tDC) that may generate regulatory T cells differentiation, thus leading to suppression of the immune response and parasite survival. The identification of the active salivary constituents could serve as a strategy for the development of new vaccines to control pathogen transmission.

## Introduction

Leishmaniasis is a vector-borne disease transmitted exclusively by sand fly bites in which the host is inoculated with saliva and regurgitated parasites during the blood meal [1]. There is evidence that Phlebotomine saliva enhances the infectivity of many different *Leishmania* species [2–5]. This can be attributed to numerous substances within the saliva with pharmacologic properties that induce vasodilatation, anticoagulation, anti-inflammation, and immunoregulation [6]. These effects are associated with the capacity to selectively inhibit several macrophage functions, including Ag presentation and NO and hydrogen peroxide production, thus inhibiting the ability of macrophages to kill intracellular *Leishmania major* [7–13]. Furthermore, in naïve animals or those not previously exposed to sand fly bites, vector saliva inhibits production of protective type 1 cytokines such as IL-12 and IFN -γ [3,14,15] while it enhances production of IL-10, IL-4, IL-6, and PGE_2_—all of which enhance the survival of *Leishmania* parasites [16–18]. Thus, identification of active salivary constituents could help to prototypes for use in development of vaccine strategies to control pathogen transmission.

We are currently isolating bioactive compounds from saliva of several bloodfeeding arthropods including Phlebotomine vectors. Systemic pre-treatment of mice with salivary gland extracts (SGE) ^3^ from the Old World species *Phlebotomus papatasi* and *Phlebotomus duboscqi* inhibited neutrophil migration during OVA-induced immune peritonitis [19]. By exploring the specific mechanisms of saliva activity, we found that Phlebotomine saliva acted preferentially on APCs and thus inhibited the ability of dendritic cells (DCs) to present Ags to T cells. These anti-inflammatory effects seemed to depend on the sequential production of PGE_2_ and IL-10 by DCs, as both cytokines acted in an autocrine manner [19]. *P*. *papatasi* SGE could therapeutically control collagen-induced arthritis pathogenesis [20]. Adenosine (ADO) and AMP were purified and identified as the active pharmacologic components in SGE responsible for immunomodulatory activity. Indeed, ADO and AMP act preferentially on DCs to block their Ag-presentation function, which interferes with Th17 cell activation and consequently suppresses the inflammatory immune response [20].

DCs are key cells in induction of immune responses to *Leishmania* by acting as both host cells and APCs, modulating specific cellular immune responses and, after appropriate activation, also operating as effector cells for intracellular parasite killing [21–23]. DC-produced cytokines such as IL-1, TNF-α, and IL-12p40 are needed for immune responses and appropriate control of *Leishmania* infections [24,25]. Moreover, release of mediators such as IL-10 and TGF-β by DCs and IL-4 by T lymphocytes might promote survival and multiplication of parasites in infected cells [26,27]. Interestingly, ADO has a broad range of effects on inflammatory leukocytes, including DCs: ADO downregulates production of pro-inflammatory mediators and expression of costimulatory markers, which diminish DC capacity to initiate and amplify inflammatory immune responses [28]. Thus, it is plausible that nucleosides present in *P*. *papatasi* SGs could play a central role in the establishment of *Leishmania* infections by modulating DC function.

In the current study, we demonstrate that ADO and AMP in the same amounts found in a single pair of *P*. *papatasi* salivary glands facilitate establishment of *Leishmania amazonensis* infection in the vertebrate host. The exacerbative effect was strictly associated with generation of tolerogenic DCs (tDCs) and induction of regulatory profile in effector T cells (Teffs) through an A2_A_R-dependent mechanism.

## Methods

### Ethics statement

All experiments were conducted in accordance with the National Institutes of Health (NIH) guidelines on the welfare of experimental animals and with the approval of the Ethics Committee of the School of Medicine of Ribeirão Preto (Number 196/2011).

### Mice

Female C57BL/6 (wild type; WT), C57BL/6-IL-10^-/-^, BALB/c, and BALB/c-A2_A_R^-/-^mice, 18–22 g in weight, were housed in the animal facility of the Department of Biochemistry and Immunology, School of Medicine of Ribeirão Preto, University of São Paulo (Brazil), in temperature-controlled rooms (22°–25°C) and received water and food ad libitum.

### Parasite inoculation, lesion measurement, and parasite load estimation

Stationary-phase promastigote forms of *Leishmania amazonensis* (10^6^ parasites or for some infections 10^3^ parasites) or *Leishmania major* (10^6^ parasites) were diluted in 10 μl of a mixture containing 1 nmol of ADO plus 1 nmol of AMP (both from Sigma, St. Louis, MO) in PBS, which are similar amounts to those present in the extract from one pair of *P*. *papatasi* SGs [20]. In some experiments, mice were infected with parasites in the presence of SGE diluted in PBS that was or was not pretreated with adenosine deaminase (ADA; 4.3 U; Sigma). Ear lesion size—defined as the difference in thickness between the infected ear and the non-infected contralateral ear—was monitored weekly using digital calipers (Mitutoyo, Suzano, SP, Brazil). Parasite load was determined by quantitative limiting dilution assay as previously described [29].

### Cell isolation from lesions

Ears from infected mice were collected and incubated at 37°C for 1 h in RPMI-1640 medium with 2 mM of L-glutamine, 100 U/ml of penicillin, 100 μg/ml of streptomycin (all from Gibco, Grand Island, NY) and 500 μg/ml of liberase CI (Roche, Basel, Switzerland). Tissues were processed in Medcons by a Medimachine (both from BD Biosciences, San Diego, CA). After processing, the cells were filtered through a 50-μm filter, viability was assessed by trypan blue exclusion, and cell concentrations were adjusted (1x 10^6^ cells/ tube).

### Flow cytometry

Immunostaining was performed with anti-CD3, anti-CD4, and anti-CD25 Abs conjugated to FITC, PE, or PerCP fluorochromes. For regulatory T cell (Treg) phenotyping, CD4^+^CD25^+^ cells were stained with anti-FoxP3, anti-CD103, anti- CD39, and anti-CD73 Abs conjugated to PECy7, APC, or Alexa700. For intracellular staining, cells were permeabilized with a Cytofix/ Cytoperm kit (BD Biosciences) according to the manufacturer’s instructions. For in vivo analyses of DC maturation, cells were harvested, stained with CD11c and MHC class-II Abs, conjugated to Alexa488 and PE or control isotypes, and characterized by flow cytometry to determine surface expression profiles. For all analyses, the results were compared to those obtained with cells stained with isotype control Abs (all Abs were from BD Biosciences and eBiosciences, San Diego, CA). Cell acquisition (~ 2 x 10^5^ cells / tube) was performed on a FACSort flow cytometer with CellQuest software (BD Biosciences). Data were plotted and analyzed with CellQuest and FlowJo (Tree Star, Ashland, OR) software.

### Cell cultures

Single-cell suspensions of draining retromaxillary lymph nodes (LNs) were prepared aseptically, diluted to a concentration of 2 × 10^6^ cells/ml, and dispensed into 48-well plates in a total volume of 500 μl of complete RPMI-1640 medium (1 × 10^6^ cells/well; Gibco) with or without soluble *Leishmania* Ag (5 μg/ml). Cell culture supernatants were harvested after 72 h of culture at 37°C in 5% CO_2_, and levels of IL-10 in the supernatants were determined by ELISA with commercial kits (BD Biosciences and R&D Systems, Minneapolis, MN). For the co-culture assays, CD4^+^CD25^-^or CD4^+^CD25^+^ cells from the draining LNs of the nucleoside- or PBS-treated groups were isolated using a CD4^+^CD25^+^ Regulatory T cell kit (Miltenyi Biotec, Auburn, CA) in accordance with the manufacturer’s instructions, and a purity of ~ 95% was obtained for each T subset. For the *in vitro* co-culture assays, CD4^+^CD25^+^ cells were added to or not wells of CD4^+^CD25^-^cells at a ratio of 5:1 (CD4^+^CD25^-^: CD4^+^CD25^+^); the wells were subsequently stimulated with plate-bound α-CD3 (2 μg/ml) plus α-CD28 (1 μg/ml) or incubated in medium alone for 96 h in a total volume of 200 μl per condition. The supernatants were harvested to measure IL-10 production.

### BMDC generation

Bone marrow-derived cells (BMDC) were isolated from 6- to 8-wk-old C57BL/6 naïve mice and cultured with murine GM-CSF (20 μg/ml; Peprotech, Rocky Hill, NJ). On d 3, half of the supernatant was gently removed and replaced with the same volume of supplemented medium. On d 6, non-adherent cells were collected and positively selected with anti-CD11c magnetic beads according to the manufacturer’s instructions (Miltenyi Biotec) to eliminate residual macrophage and granulocyte contamination. Flow cytometric evaluation of the purified BMDCs showed that 90% of cells expressCD11c^interim or high^.

### Effect of nucleosides on *L*. *amazonensis*-infected BMDC

BMDCs (1 × 10^6^ /ml) were incubated in RPMI-1640 supplemented with 10% FBS with or without ADO+AMP for 1 h. The cells were subsequently infected with GFP-expressing promastigote forms of *L*. *amazonensis* (1 × 10^7^ parasites/ml). Supernatants were collected to measure TNF-α and IL-10 production by ELISA.

### BMDC-dependent Treg differentiation assay

BMDC treated with ADO+AMP or medium for 3 h were stimulated overnight with LPS (50 ng/ml) and then cultured with freshly isolated naïve CD4^+^CD25^-^cells in the ratio of 1:10 (DC:lymphocytes) under polarizing conditions: rmTGF-β (5 ng/ml), rmIL-2 (100U/ml), anti-IFN-γ (10 μg/ml) and anti-IL-4 (10 μg/ml) at 37°C in 5% CO_2_ for 7 d. As differentiation control, natural Tregs (nTregs) (CD4^+^CD25^+^) or Th0 (CD4^+^CD25^-^) were cultured in the presence of IL-2 (100 U/ml) for T cell maintenance. Lymphocytes were then washed and phenotyped for expression of surface markers using mAb-specific against CD39, CD73, CD103 and FOXP3 (BD Biosciences and eBioscience).

### Quantitative RT-PCR

Total RNA was isolated from ears of mice co-inoculated with ADO+AMP plus parasites at wk 11 post infection (p.i.) or BMDCs pre-incubated with ADO+AMP after 24 h of stimulation with *L*. *amazonensis* (5 parasites:1 cell) using the Illustra RNAspin Mini (GE Healthcare, Buckinghamshire, UK). Gene expression was normalized to expression of the GAPDH gene. COX_2_ primer sequences are as follows: GAPDH forward: 5ˊ-TGCAGTGGCAAAGTGGAG AT-3ˊ, reverse: 5ˊ-CGTGAGTGGAGTCATACTGGAA-3ˊ; COX_2_ forward: 5ˊ-GTGGAAAAA CCTCGTCCAGA-3ˊ, reverse: 5ˊ-GCTCGGCTTCCAGTATTGAG-3ˊ; IL-10 forward: 5ˊ-TGG ACAACATACTGCTAACCG-3ˊ, reverse: 5ˊ-GGATCATTTCCGATAAGG CT-3ˊ; TGF-β forward: 5ˊ-ACCGCAACAACGCCATCTAT-3ˊ, reverse: 5ˊ-TCAAAAGCAAGCCACTCA GGC-3ˊ; and IDO forward: 5ˊ-AAGCAATCCCCACTGTATCC-3ˊ, reverse: 5ˊ-CAATGCTTT CAGGTCTTGACG-3ˊ. To quantify A2_A_ receptor (A2_A_R) and A2_B_R expression, total mRNA was extracted from DC culture harvested 24 h p.i.. A2_A_R forward: 5ˊ-TTCTTCGCCTGCTTT GTCCT-3ˊ, reverse: 5ˊ-ATACCCGTCACCAAG CCATT-3ˊ; and A2_B_R forward: CTGCTC ATAATGCTGGTGATCT, reverse: ATCAGTTCCATGCGCTGA.

### Statistical analysis

Data are expressed as the mean ± SEM and are representative of 2–4 independent experiments. Results from individual experiments were not combined because they were analyzed individually. The means from the different groups were compared by analysis of variance (ANOVA) followed by Tukey's honest significant difference (HSD) test. Statistical significance was set at *p*<0.05.

## Results

### ADO and AMP exacerbate *Leishmania sp* infection

**Fig 1:**
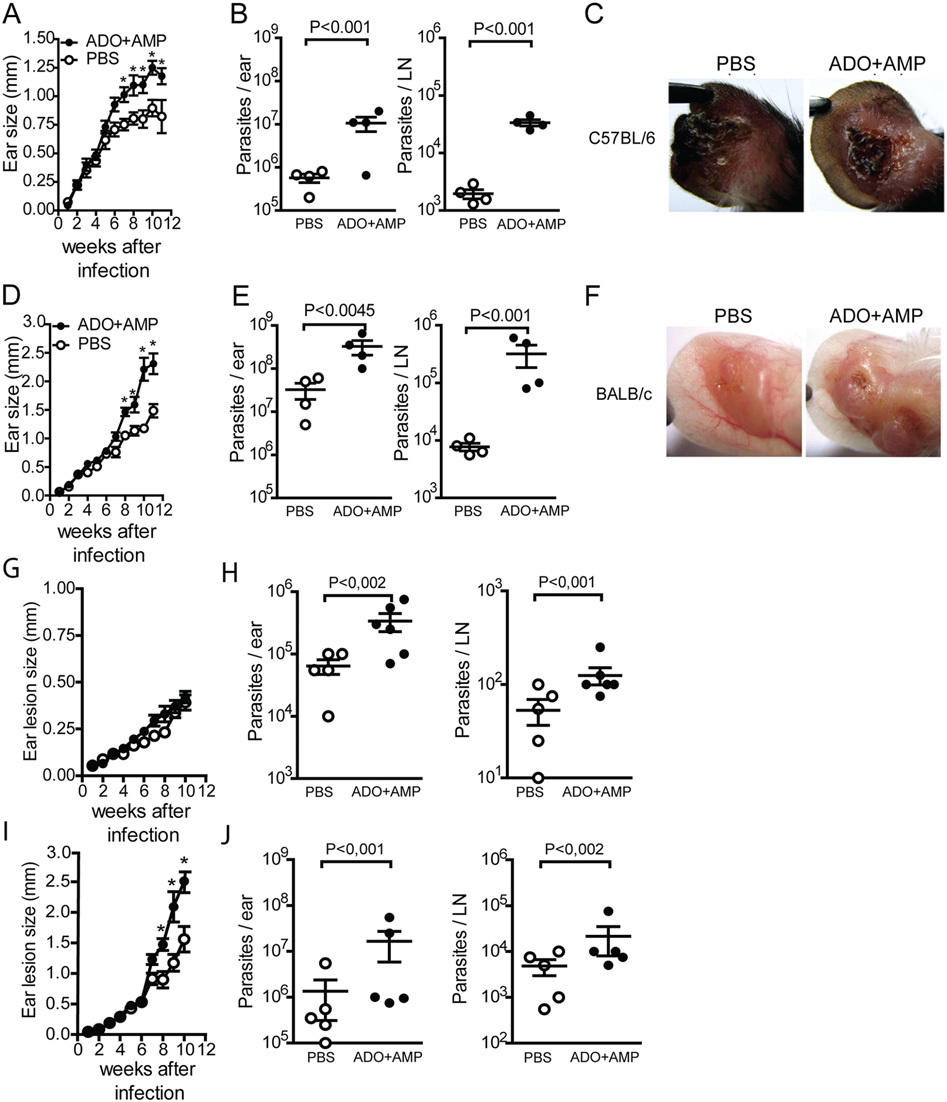
ADO+AMP exacerbates *Leishmania amazonensis* infection in vivo. C57BL/6 or BALB/c mice were infected with 10^6^ *L*. *amazonensis* promastigotes in the presence of ADO+AMP (●) or PBS (Ο). The infection course was monitored weekly by measuring sizes of ear lesions with metric calipers. In *A* and *D*, lesion size was determined by the difference in thickness between the infected ear and the opposite uninfected ear on each mouse. Measurements are reported in millimeters (mm); *n* = 4 mice per group. Data are shown as the mean ± SEM and are representative of three different experiments. *, *p*<0.05 relative to the PBS-treated group. Parasite burdens in ears (*B*) and draining lymph nodes (*E*) at 11 weeks post infection were determined by a limiting-dilution assay. Data are shown as the means ± SEM of three separate experiments; each experiment was performed with five mice per group *n* = 5. *C* and *F* are representative photographs of mouse ears at 11 weeks post infection. *In* G and H, BALB/c mice were infected with 10^3^ metacyclic promastigotes forms of *L*. *amazonensis* in the presence of ADO+AMP (●) or PBS (Ο). In I-J, as in D–E, but using infection with 10^6^ stationary-phase *L*. *major* promastigotes. Data are shown as the means ± EPM performed with five to six mice per group *n* = 5–6.

To investigate whether ADO and AMP present in *P*. *papatasi* saliva are constituents that may exacerbate leishmaniasis, C57BL/6 and BALB/c mice were intradermally infected in the ear with 1 × 10^6^ promastigote forms of *L*. *amazonensis* in the presence or absence of equimolar amounts of ADO and AMP present in one pair of SGs. As reported by Ribeiro et al. [30], in this salivary extract, ADO and AMP are detected on the order of 1 nmol per pair of glands. Co-inoculation of parasites with nucleosides exacerbated infection in both strains of mice when compared with the control group (inoculated with parasite plus PBS) (Fig 1). Animals co-inoculated with parasites and nucleosides showed a significant increase in ear thickness and ulcerative lesion starting at week 8 p.i. (BALB/c, *p*<0.045; C57BL/6, *p*<0.01) (Fig 1A and 1D) that progressed until the animals’ deaths at wk 12 p.i. (Fig 1C and 1F). The number of parasites present in the ear lesion, as well as in draining LNs, was also greater in the group co-inoculated with parasite and nucleosides compared with the group co-inoculated with parasite and PBS (Fig 1B and 1E).

To determine the potential effect of nucleosides on the establishment of *L*. *amazonensis* infection, we infected BALB/c mice with low numbers of *L*. *amazonensis* (10^3^ promastigote forms) in the presence or absence of adenosine and AMP to mimic the natural model of infection. Lower numbers of parasites promoted reduced and delayed lesion development in mice coinoculated with ADO+AMP or PBS over time (Fig 1G). Despite similar rates of edema in both groups (ADO+AMP and PBS), mice that received nucleosides showed higher parasite titers in the ears and lymph nodes than mice inoculated with parasites in PBS (Fig 1H). Our data are consistent with a previous study showing that *Lutzomyia longipalpis* SGE maintains the persistence of *L*. *braziliensis* within the skin without interfering with lesion size during low-dose infection [31].

*P*. *papatasi* is not a natural vector of *L*. *amazonensis*, but it is transmitted by the *Lutzomyia* genus and does not contain salivary nucleosides [30]. To address the impact of nucleosides on species that are normally transmitted by *Phlebotomus papatasi*, we infected BALB/c mice with *L*. *major* (10^6^ parasites / mice) and adenosine+AMP. The mixture of nucleosides promoted the exacerbative effect of saliva on *L*. *major* infection. During the first 6 weeks after infection, the ear lesions were similar between PBS and ADO+AMP coinoculated mice. Afterward, the lesions progressed in both groups, but they were clearly pronounced in the group that was coinoculated with ADO+AMP (Fig 1I). The larger lesions found in nucleoside coinoculated mice were associated with impaired control of parasite growth; this group presented higher parasite loads in both ear lesions and draining lymph nodes at the 10^th^ wpi (Fig 1J). Thus, these data suggest that the amounts of ADO and AMP in one pair of SGs of *P*. *papatasi* are sufficient to establish cutaneous *Leishmaniasis* causing species.

**Fig 2:**
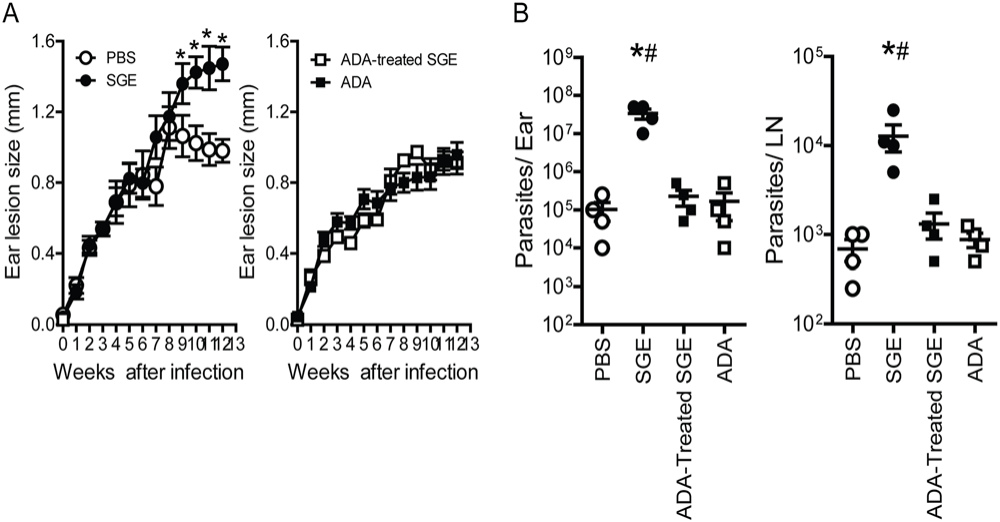
Deamination of salivary nucleosides reversed the immunosuppressive effect of salivary gland extract (SGE). C57BL/6 mice were infected with *Leishmania amazonensis* (1 × 10^6^ parasites/ear) in the presence of PBS (Ο) or SGE (1 gland/ear) (●). In some groups, SGE (■) or PBS (□) had been previously incubated with ADA (4.3 U) for 3 h before infection. *A*, Ear lesion growth course was determined by difference in thickness between the infected and opposite uninfected ear for each mouse. Measurements are reported in millimeters (mm), and *n* = 4 mice per group. Parasite burdens in the ears and draining lymph nodes (LN) (*B*) at 12 weeks post infection were determined by a limiting-dilution assay. Data are shown as the means ± SEM of three separate experiments; each experiment was performed with five mice per group (*n* = 5). *, *p*<0.05 relative to the PBS-treated group; ^#^, *p*<0.05 compared with the ADA or ADA-SGE-treated groups.

To verify whether both nucleosides are the salivary compounds responsible for vector-induced establishment of infection, SGEs (1 pair of glands/ear) from *P*. *papatasi*—previously treated or not with ADA, an enzyme that catabolizes ADO [20,32]—were co-inoculated with the *Leishmania* parasite. As control groups, ADA or PBS was co-inoculated with *L*. *amazonensis*. We found that the sizes of lesions were significantly larger in mice co-inoculated with parasite plus SGE compared with those that received parasite plus PBS control (Fig 2A) and that they were correlated with the numbers of parasites present in the ear and draining LNs (Fig 2B). Treatment of SGEs with ADA abolished the exacerbative effect of SGEs during *L*. *amazonensis* infection, resulting in reduced ear lesions (Fig 2A) as well as reduced parasite numbers in the ear and draining LNs (Fig 2B). In addition, no differences were observed either in lesion or parasite burden among ADA, SGE-treated ADA, or PBS groups.

### Nucleosides induce immunoregulatory molecules during *L*. *amazonensis* infection

**Fig 3:**
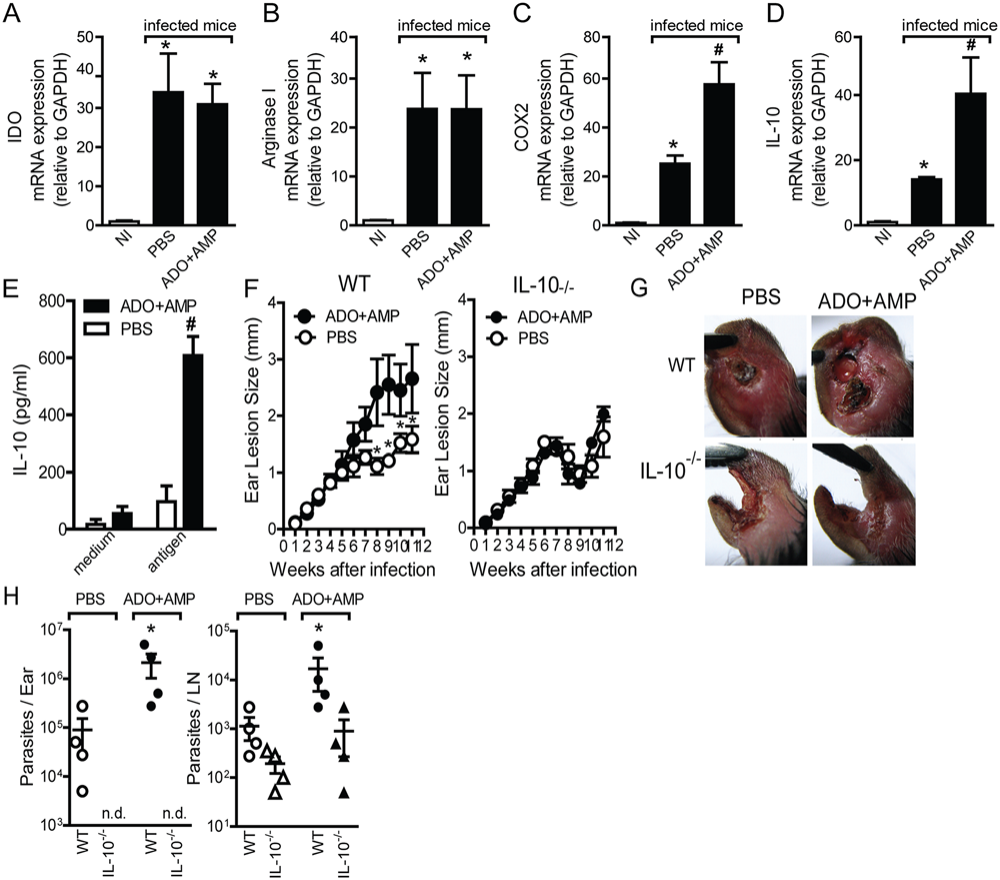
Nucleosides modulate *in vivo* expression of suppressive genes and mediate immunoregulatory effects by IL-10 during *Leishmania amazonensis* infection. Expression levels of IDO (*A*), arginase-1 (*B*), COX_2_ (*C*), or IL-10 (*D*) mRNA into ears from C57BL/6 mice that were infected or not (naïve) with 10^6^ *L*. *amazonensis* promastigotes in the presence of ADO+AMP or PBS at 11 weeks post infection. *E*, IL-10 production in cultured draining lymph node cells from ADO+AMP or PBS-treated mice was determined by ELISA. Data are shown as the means ± SEM of two separate experiments; each experiment was performed with four (naïve) or six (infected) mice per group (*n* = 4–6). *, *p*<0.05 relative to naïve mice; ^#^, *p*<0.05 compared with the PBS-treated group. Ear lesion (*F*, *G*) and parasite burden (*H*)—by a limiting-dilution assay—in WT and IL-10^-/-^mice at 11 weeks p.i. were determined. Data are shown as the means ± SEM of two separate experiments; each experiment was performed with five mice per group (*n* = 5). *G*, representative photographs of ears at 11 weeks post infection.

Expression of IDO, arginase 1, COX_2_, and IL-10 have been reported to play a key mechanism that triggers several immunosuppressive effects and can be induced by ADO [20,28]. We therefore examined whether such factors are modulated by nucleosides during infection. The results reveal higher mRNA expression of IDO, arginase 1, COX_2_, and IL-10 for the ears of mice infected with parasites and PBS compared with those of uninfected mice (Fig 3). Furthermore, while IDO (Fig 3A) and arginase 1 (Fig 3B) expression levels were similar, COX_2_ (Fig 3C) and IL-10 (Fig 3D) mRNA levels were upregulated in ears of mice co-inoculated with parasite and ADO+AMP. Enhancement of mRNA for COX_2_ and IL-10 was 2-fold and two- to three-fold, respectively (Fig 3C and 3D).

We previously demonstrated that ADO—already present in saliva and/or generated by AMP metabolism by CD73 expressed in DCs—most likely accounts for most, if not all, anti-inflammatory activity presented by *P*. *papatasi* SGEs through a mechanism dependent on PGE_2_-induced IL-10 release [20]. In addition, IL-10 mRNA was upregulated in ears of mice infected with parasite plus nucleosides (Fig 3D). Attempting to address the role of IL-10 in exacerbation of infection induced by nucleosides, we measured production of IL-10 in culture supernatant of total cells from draining LNs of C57BL/6 co-inoculated with parasites plus ADO+AMP or PBS and re-stimulated them in vitro with soluble *Leishmania* Ag (SLA). Stimulation with SLA did not induce significant amounts of IL-10 in culture supernatant of draining LN cells from mice co-inoculated with PBS, compared with control (medium) (Fig 3E). In contrast, the supernatant of draining LN cells from mice co-inoculated with parasites and nucleosides showed high levels of IL-10 after SLA stimulation, compared with the PBS-treated group (Fig 3E). Co-inoculation of parasites and ADO+AMP in IL-10^-/-^mice resulted in lack of exacerbative effect by nucleosides during *L*. *amazonensis* infection, as observed by the reduction of lesion size (Fig 3F and 3G) and a decrease in the number of parasites present in the ear and draining LNs (Fig 3H). Interestingly, despite the fact that infected IL-10^-/-^mice showed reduced ear lesion size, they developed a severe ulcerative and necrotic lesion even in the presence or absence of nucleosides (Fig 3G), suggesting that the lack of regulation of the immune response induced by IL-10 favors ear cartilage destruction due to excessive inflammatory response triggered during infection by *L*. *amazonensis*. In fact, we did not detect parasites in the ears of IL-10^-/-^mice with or without nucleosides (Fig 3H). Together, our data suggest that IL-10 released at the site of infection strongly contributes to exacerbative activity of nucleosides during *Leishmania* infection.

### Nucleosides induce regulatory markers in effector T lymphocytes

**Fig 4:**
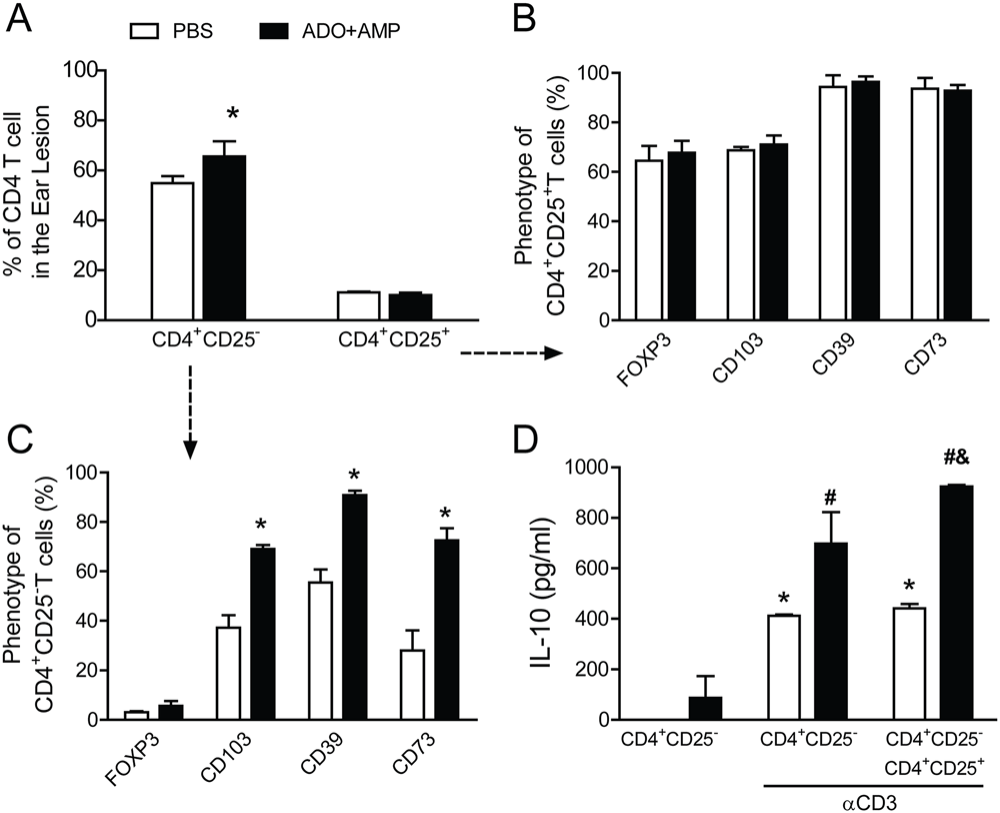
Salivary nucleosides induce expression of a regulatory T cell profile in effector T cells. Ear cells from mice infected with *Leishmania amazonensis* in the presence of ADO+AMP (■) or PBS (□) were harvested at 11 weeks post infection. *A*, Lymphocytes from the ears were phenotyped by flow cytometry with FITC-conjugated anti-CD3, PerCP-conjugated anti-CD4, and PE-Cy7-conjugated anti-CD25 Lymphocytes were selected with CD4^+^CD25^+^ (*B*) or CD4^+^CD25^-^ (*C*) gates, and these populations were subsequently analyzed for expression of FoxP3, CD39, CD73, and CD103. CD4^+^CD25^-^lymphocytes (2 × 10^6^ cells/ml) were isolated from draining lymph nodes of PBS- (□) or ADO+AMP- (■) infected mice at 8 weeks post infection. The lymphocytes were re-stimulated in vitro with αCD3 (2 μg/ml) plus αCD28 (1 μg/ml) for 96 h. CD4^+^CD25^+^ cells were added to some of the CD4^+^CD25^-^culture wells. *D*, Suppressive activity was determined by ELISA to measure IL-10 secretion in the culture supernatants. Data are expressed as the means ± SEM and are representative of two independent experiments that were each performed with four mice per group (*n* = 4). ^#^, *p*<0.05 relative to the PBS-treated group.

Further investigating the mechanism by which nucleosides exacerbated *L*. *amazonensis* infection, we evaluated the phenotype of T cells isolated from the ears of mice inoculated with parasites and ADO+AMP or PBS. Nucleoside treatment did not interfere in expression of CD4^+^ T cells compared with the control group (Fig 4A). A similar effect was observed regarding the CD4^+^CD25^+^ population (Fig 4A). Expression of Treg phenotypes such as FoxP3, CD103, CD39, and CD73 (Fig 4B) in the CD4^+^CD25^+^ cell population was likewise similar in both groups. Unexpectedly, expression of markers characteristic of Tregs in the CD4^+^CD25^-^population was significantly increased, as observed by the higher expression of CD103, CD39, and CD73 in nucleoside-treated animals compared with the PBS-treated group (Fig 4C, S1 Fig). These data suggest that nucleosides from *P*. *papatasi* saliva may induce the Treg phenotype in effector T lymphocytes. Because salivary nucleosides potentiated IL-10 production (Fig 3D and 3E) and mediated susceptibility to the infection (Fig 3F–3H), we addressed whether the CD4^+^CD25^-^expressing Treg markers in the CD4^+^CD25^-^population could contribute to nucleoside-induced IL-10 production. Therefore, purified CD4^+^CD25^-^T cells from draining LNs were cultured with plate-bound αCD3 (2 μg/ml) plus αCD28 (1 μg/ml) or medium with or without CD4^+^CD25^+^. As expected, CD4^+^CD25^-^T cells from infected mice stimulated with plate-bound anti-CD3 induced showed enhanced production of IL-10 (Fig 4D). Furthermore, the culture supernatant from CD4^+^CD25^-^T cells from mice infected and treated with nucleosides produced higher levels of IL-10 after polyclonal stimulation than cultures that lacked nucleosides (Fig 4D). The addition of autologous CD4^+^CD25^+^ cells to CD4^+^CD25^-^ cultures potentiated IL-10 production when the cells were derived from the nucleoside group but not when they were isolated from the PBS group. These observations indicate that iTregs may contribute to the immunosuppressive effects of nucleosides through IL-10 release.

### Nucleoside induces a tolerogenic profile in DCs during *L*. *amazonensis* infection

**Fig 5:**
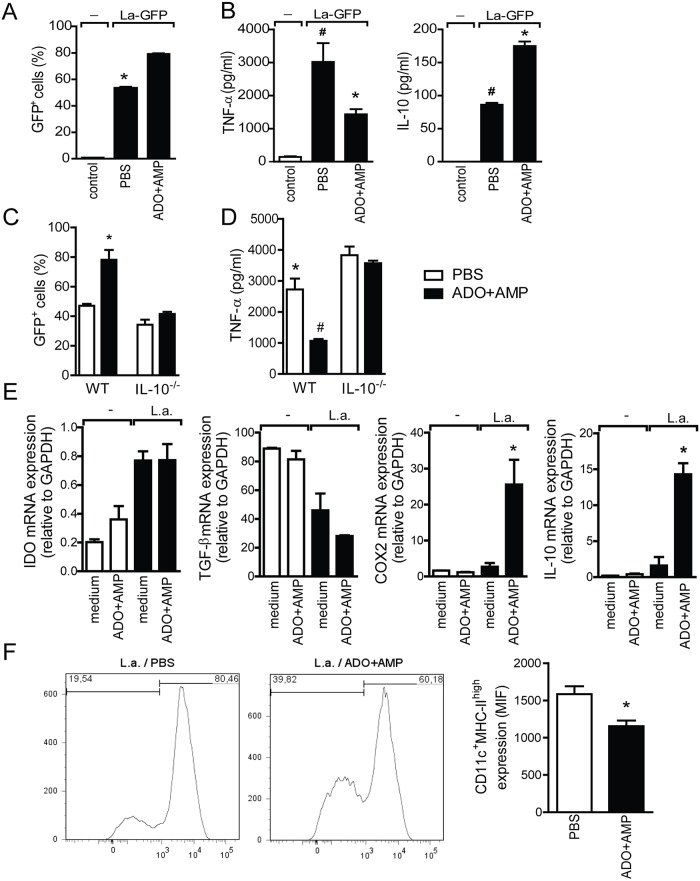
In vitro and in vivo effects of ADO+AMP on dendritic cells (DC) during *Leishmania amazonensis* infection. Bone marrow-derived cells (BMDC) from wild type or IL-10^-/-^mice were incubated without (□) or with (■) ADO+AMP for 1 h prior to infection with GFP-expressing *L*. *amazonensis*. Parasite growth in BMDCs was determined by GFP positivity and measured by flow cytometry. *A*/*C*, Bars display the relative GFP positivity and are representative of three independent experiments that were each performed in quadruplicate (*n* = 4). ^#^, *p*<0.05 relative to uninfected cells; *, *p*<0.05 relative to the PBS group. *B/D*, Levels of cytokines TNF-α and IL-10 in the culture supernatants were measured by ELISA. BMDCs (1x 10^6^ cells/ml) were incubated ± ADO+AMP for 1 h. *E*, Cells were harvested 24 h after *L*. *amazonensis* infection for quantification of IL-10, COX_2_, TGF-β and IDO mRNA expression. Data are shown as the means ± SEM from one of three independent experiments that were performed in quadruplicate (n = 4 per group). ^#^, *p*<0.05 relative to the control group; *, *p*<0.05 relative to the parasite-infected group. PBS- or ADO+AMP-infected mice were euthanized at 8 wk post infection, the draining lymph nodes were harvested, and the cells were labeled with FITC-conjugated anti-CD11c or PE-conjugated anti-MHC class II mAbs to detect DC surface markers. *F*, Representative histograms of DCs from PBS- and ADO+AMP-infected mice are shown in each box and bars display the relative mean fluorescence intensity, and data are shown as the means ± SEM; *n* = 5. ^#^, *p*<0.05 relative to naïve mice; *, *p*<0.05 relative to the ADO+AMP group.

We also evaluated the in vitro effect of nucleosides on the replicative ability of parasites when cultured with DCs. In the presence of ADO+AMP, parasite growth was enhanced (Fig 5A). The increase was approximately 33% compared to the control (PBS) group (Fig 5A). Moreover, production of pro-inflammatory mediators such as TNF-α was reduced, whereas production of IL-10 was enhanced in cultured DCs infected with the parasite in the presence of nucleosides when compared with the PBS–treated group (Fig 5B). Conversely, the exacerbative effect of salivary nucleosides in neither parasite growth (Fig 5C) nor the inhibitory effect of TNF (Fig 5D) were observed in IL-10^-\-^mice. As several factors—including IL-10, TGF-β, IDO, and PGE_2_—might modulate DC function by promoting differentiation into a tolerogenic profile [33,34], we evaluated expression of factors related to a tolerogenic profile such as IDO, TGF-β, IL-10, and COX_2_. Administration of nucleosides in BMDC culture significantly increased levels of COX_2_ and IL-10 mRNA expression (Fig 5E), which correlated with in vivo data (Fig 3C and 3D). This increase was approximately 89% for COX_2_ and 88% for IL-10 when compared with the control group (infected with parasite only). In contrast, IDO levels were not changed, and TGF-β mRNA levels were downmodulated during infection independently of presence of nucleosides. Furthermore, DCs isolated from draining LNs from mice co-inoculated with nucleosides and parasites exhibited an immature phenotype, showing a reduction in percentage and numbers of MHC-II molecules on the surface of CD11c^+^ cells compared with the PBS control group (Fig 5F; S1 Fig).

### Tolerogenic Dendritic Cells (tDCs) induced by nucleosides promote the generation of iTregs

**Fig 6:**
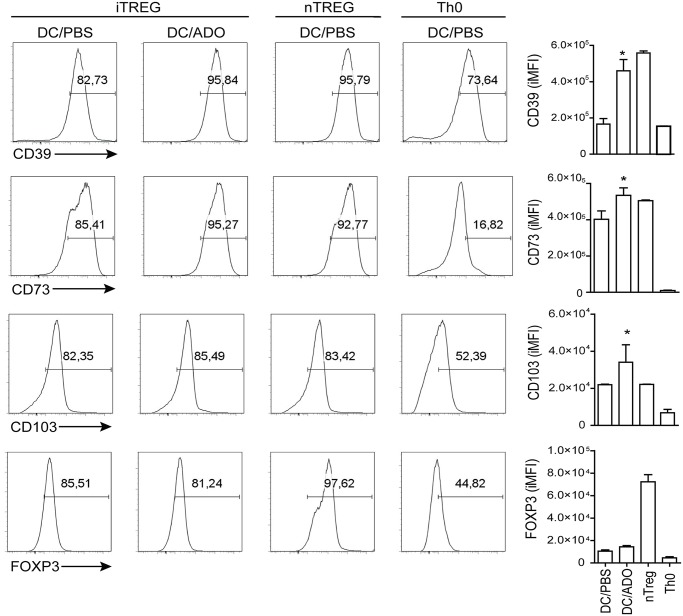
tDC generated by nucleosides present ability to induce regulatory profile on CD4^+^CD25^-^ cells. Bone marrow-derived cells (BMDC) treated with ADO or PBS and stimulated with LPS (50 ng/ml) were co-cultured with CD4^+^CD25^-^under Treg polarizing conditions at the ratio of 1:10 (BMDC:T cells). Natural Treg (nTreg, CD4^+^CD25^+^) or Th0 (CD4^+^CD25^-^) were used as positive and negative differentiation control. Representative histograms of CD39, CD73, CD103, and FoxP3 are shown in each box. Bars display the relative mean fluorescence intensity, and the results are expressed as the mean ± SEM obtained from one of three independent experiments made in triplicate (*n* = 3 per group).*, *p*<0.05 relative to the BMDC/PBS group.

We further tested whether tDCs generated by nucleosides have the potential to induce Tregs. As expected, under Treg-polarizing conditions, CD4^+^CD25^-^cultured with BMDC/PBS upregulated their CD39, CD73, CD103, and FoxP3 expression when compared with Th0 cells. Interestingly, the proportion of CD39, CD73, and CD103 was found to increase without affecting FOXP3 expression when CD4^+^CD25^-^cells were cultured with BMDC/ADO (Fig 6). The enhancement was similarly likewise observed on nTregs that expressed not only CD39, CD73, and CD103 but also FoxP3 (Fig 6). Altogether, the data suggest that nucleosides may modulate tDCs capable of inducing iTreg generation.

### The immunoregulatory effect of nucleosides is triggered though A2_A_R pathway

**Fig 7:**
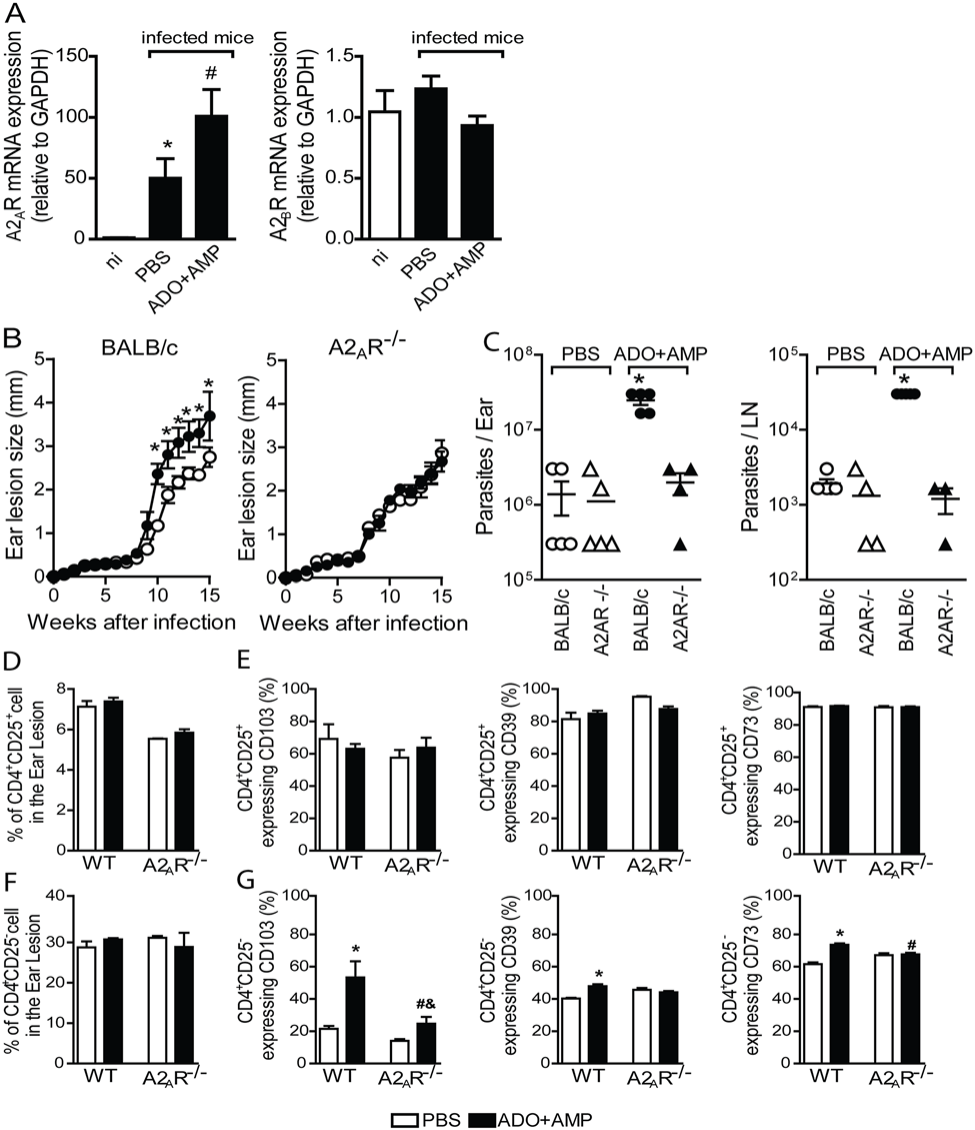
Salivary nucleosides induce iTreg in an A2_A_R-dependent manner. In *A*, ears from mice infected with *Leishmania amazonensis* (10^6^ parasites/ ear) in the presence of ADO+AMP (■) or PBS (□) were harvested at 11 weeks post infection for quantification of A2_A_R and A2_B_R. *A*, mRNA expression. Data are shown as the means ± SEM from one of two independent experiments that were each performed with four mice per group (*n* = 4 per group). *, *p*<0.05 relative to naïve mice. ^#^, *p*<0.05 relative to the PBS group. Ear lesion (*B*) and parasite burden in ear and draining lymph nodes (*C*) by a limiting-dilution assay in WT and A2_A_R^-/-^mice at 11 weeks post infection were determined. Expression levels of CD39, CD73, and CD103 (*E* and *G*) analyzed in CD4^+^CD25^+^ (*D*) and CD4^+^CD25^-^ (*E*) population. Data are shown as the means ± SEM of two separate experiments; each experiment was performed with five (burden parasite and flow cytometry) to ten (ear lesion) mice per group (*n* = 5–10).

Among ADO receptors, A2_A_R and A2_B_R mediate immunosuppressive effects by coupling to a G-protein and activating adenylyl cyclase, thereby generating the second messenger cyclic AMP that downregulates host cell activation [35]. Thus, mRNA levels of A2_A_R and A2_B_R were analyzed in the ears of infected mice. Transcripts for A2_A_R were upregulated in the ears of mice infected only with parasites and were highly expressed in ears of mice co-inoculated with parasite plus nucleosides (Fig 7A). The transcript profile of A2_B_R mRNA was not altered in either the nucleoside- or PBS-treated group, suggesting that ADO mediates immunosuppressive action through the A2_A_R.

To further examine the role of A2_A_R on the suppressive effect of salivary nucleosides, we infected mice lacking A2_A_R with *L*. *amazonensis* in the presence of ADO+AMP or PBS. Absence of A2_A_R abrogates the exacerbative effect of nucleosides on mice during disease, as observed by ear lesion development (Fig 7B) and harboring fewer parasites in lesion and draining lymph node (Fig 7C). In addition, no changes were observed in lesion or parasite burden among BALB/c-PBS, A2_A_R^-/-^-PBS, or A2_A_R^-/-^-ADO+AMP groups (Fig 7B and 7C). The lack of an exacerbative effect of nucleosides in the A2_A_R^-/-^group was followed by a committed induction of Treg markers in the CD4^+^CD25^-^population. While a slight reduction of CD73 and CD39 expression was observed in A2_A_R^-/-^-ADO+AMP compared with BALB/c-ADO+AMP, there was a remarkable decrease in CD103 expression, although that remained enhanced compared with A2_A_R^-/-^-PBS (Fig 7G). Interestingly, the percentage of the CD4^+^CD25^+^ subset was reduced in A2_A_R^-/-^independently of nucleosides (Fig 7D), suggesting the involvement of A2_A_R signaling on nTreg generation (35).

## Discussion

Several studies have shown that sand fly saliva plays a key role in the establishment of *Leishmania* infections in vertebrate hosts through inhibition of several immune functions [1]. Among the pharmacologic substances involved in this inhibition, we recently identified ADO and AMP as the *P*. *papatasi* saliva constituents that inhibit activation and function of DCs [20]. Thus, we addressed whether ADO and AMP were the *P*. *papatasi* saliva components responsible for establishment of *Leishmania* infections in vertebrate hosts. Co-inoculation of parasites with nucleosides promoted the same disease exacerbation profile as total saliva, which suggests that these could be the constituents involved in establishment of *Leishmania sp* infections. Deamination of salivary nucleosides with ADA—an enzyme that catabolizes ADO—markedly abolished the exacerbative effects of SGEs during leishmaniasis. Despite the fact that *P*. *papatasi* not being a natural vector of *L*. *amazonensis*, which is transmitted by *Lutzomyia flaviscutellata*, this vector can transmit *L*. *amazonensis* under laboratory conditions. Salivary gland extract from other species, such *P*. *papatasi* and *P*. *sergenti*, could establish *Leishmania amazonensis* infection by promoting lesions as rapidly and as large in size as those produced by *L*. *longipalpis* [36]. Furthermore, proteins from *Lutzomyia longipalpis*, LJM11 and LJM19, induce immunity against different species of *Leishmania sp* (*L*. *major*, *L*. *infantum* and *L*. *braziliensis*)[37]. Our data showed that, similar to *L*. *amazonensis* infection, adenosine and AMP also promote an exacerbative effect during *L*. *major* infection. We do not rule out the possibility of other salivary components (such as proteins, prostaglandins, etc.) that may contribute to the exacerbative role of saliva in leishmaniasis, but we believe that the strongest immunomodulatory effects of *P*. *papatasi* saliva are at least partly mediated by nucleosides.

Although different species present different salivary constituents, some anti-inflammatory properties may be similar among them. For example, the saliva from the Old World species Phlebotomines *P*. *papatasi* and *P*. *duboscqi* acts mainly on dendritic cells and induces the production of IL-10 by a mechanism dependent on PGE_2_. In turn, PGE_2_ acts in an autocrine manner to reduce the antigen-presenting ability of DCs [19]. Previous studies have also shown *in vitro* and *in vivo* examples of *Lutzomyia longipalpis* saliva promoting IL-10, PGE2 and TGF-β production by macrophages and T cells, which exacerbates *Leishmania* infection [38]. Moreover, the genetic ablation of IL-10 prevents the detrimental effect of *Lutzomyia longipalpis* SGE on both *Leishmania major* and *L*. *amazonensis* infections [39,40]. Our data showed that a significant increase in IL-10 production was observed in culture supernatants of draining LNs from animals co-inoculated with parasites and nucleosides. Furthermore, IL-10 deficiency (IL-10^-/-^mice) reversed the immunosuppressive effects of salivary nucleosides during infection; however, ablation of IL-10 promoted significant tissue damage independently of nucleoside treatment. This damage did not correlate with parasite numbers but instead resulted from an excessive inflammatory response. Studies have shown the potent anti-inflammatory effects of IL-10 by demonstrating several functions including the ability to limit tissue damage during infections and the ability to regulate the duration and intensity of immune inflammatory reactions [41]. Thus, understanding how the substances present in *P*. *papatasi* saliva, such as AMP and adenosine, are involved in the exacerbation of infection in a fully experimental model may explain the consistent exacerbative role of saliva in leishmaniasis.

Tregs limit the magnitude of effector responses against *Leishmania spp.*, which can result in a failure to properly control parasitic infections [42]. Tregs express high surface levels of CD39 and CD73, and ADO generation is a mechanism by which Tregs exert their suppressive effects [43]. NECA (a synthetic ADO analog) or an A2_A_R agonist could increase expression of Treg markers FoxP3, CD39, CD73, and CTLA-4 in the CD4^+^CD25^-^population in addition to expanding the FoxP3-, CD39-, CD73-, and CTLA-4-expressing CD4^+^CD25^+^ cell population [44]. We present evidence that the nucleosides from sand fly saliva could generate iTregs at the infection site. Co-inoculation of parasites plus nucleosides did not increase the numbers of nTregs (CD4^+^CD25^+^FoxP3^+^) but increased the levels of CD103, CD73, and CD39 expression on CD4^+^CD25^-^cells. The high surface levels of CD39 and CD73 on Teffs (CD4^+^CD25^-^) help to generate ADO in the extracellular compartment by cleaving AMP in *P*. *papatasi* saliva, thus contributing to exacerbation of *Leishmania* infections as a consequence of IL-10 production. Indeed, IL-10 was substantially produced by iTregs generated in response to nucleosides, and this phenotype was pronounced when these cells were co-cultured with autologous nTregs. iTregs could suppress the proliferation of effector T cells in a cell contact–independent fashion. Key cytokines that have been associated with the suppressive activity of iTregs include IL-10 [45] and TGF-β[46], which are crucial for continuous suppression of the effector T cells [47] that are involved in pathogen restriction, such as Th1 and Th17 [48]. IL-10 released peripherally by iTreg cells can sustain tolerance by converting naive T cells to the next generation of FoxP3^+^cells [47]. Thus, it is possible that IL-10—when secreted by salivary nucleoside-generated iTregs—contributes to the exacerbation of leishmaniasis.

iTreg generation depends on activation of conventional CD4^+^ T cells by tDCs. tDCs are characterized by low surface expression of costimulatory molecules such as MHC-II, CD80, CD86, and CD40 and high expression of CD39 [33]. tDCs promote alterations in the immune system by inducing anergy or deletion of autoreactive T lymphocytes or even by inducing Treg generation [49,50]. tDC co-cultured CD4^+^ T cells exhibited increased levels of CD25, CTLA-4, FoxP3, and CD39 expression and responded weakly when stimulated with Ag [34]. Of interest is that a similar tDC phenomenon was observed when DCs were incubated with ADO+AMP plus parasites, thus establishing a direct relationship between salivary nucleosides, Treg generation and, ultimately, the exacerbation of leishmaniasis. Previously, we demonstrated that ADO from *P*. *papatasi* SGE could upregulate CD73 surface expression and downregulate MHC-II and CD86 surface expression on DCs both in vitro and in vivo [19,20]. In the present study, DCs from draining LNs of animals co-inoculated with nucleosides and parasites exhibited a semi-mature phenotype with downregulated surface MHC-II expression and reduced production of pro-inflammatory cytokines. Furthermore, administration of ADO on DC culture promoted generation of regulatory markers on the CD4^+^CD25^-^subset.

Several factors—including IL-10, prostaglandin E_2_, TGF-β, and vitamin D3—modulate DC function and favor tDC differentiation [34,51,52]. PGE_2_, a lipid mediator synthesized by COX_2_, promotes DC-mediated production of several suppressive factors such as IL-10 and IDO [53]. Interestingly, our data show that parasite infection in the presence of nucleosides did not alter IDO and TGF-β levels but induced expression of IL-10 and COX_2_ mRNA both in vitro and in vivo. We previously reported that *P*. *papatasi* SGEs inhibit immune peritonitis by sequential production of PGE_2_ and IL-10, which acted in an autocrine manner on DC function [19]. Likewise, ADO and AMP in *P*. *papatasi* SGE exhibited anti-inflammatory activities against collagen-induced arthritis by blocking DC Ag presentation and secretion of pro-inflammatory cytokines. Strikingly, we demonstrated that ADO could enhance PGE_2_ production from LPS-stimulated BMDCs [20]. Thus, it is plausible that *P*. *papatasi* ADO-induced secretion of SGE IL-10 and PGE_2_ could induce a tDC profile, thus inhibiting DC function and ultimately contributing to establishment of an infection.

ADO effects are mediated by four surface receptors—A1R, A2_A_R, A2_B_R, and A3R—which are present on many cell types. Among these, A2_A_R and A2_B_R regulate multiple physiologic responses including the anti-inflammatory and immunosuppressive effects of ADO. In fact, genetic ablation or pharmacologic inhibition of A2_A_R or A2_B_R leads to excessive immune responses [54,55]. Here we show that A2_A_R, but not A2_B_R, was highly expressed in the ears of mice co-inoculated with parasites and nucleosides. The immunosuppressive activity of ADO during leishmaniasis is mediated through an A2_A_R-dependent-mechanism, indicated by genetic deletion of the receptor, which leads to abrogated intensification of the infectious process mediated by salivary nucleosides. This phenomenon strictly correlated with a lack of induction of Treg generation. Although we did not evaluate the sequential production of PGE_2_/IL-10 as a result of A2_A_R signaling on DCs, we have strong evidence to support this pathway. We previously reported that blocking A2_A_R with a selective antagonist (8,3,cloroesterylcafeine) prevented inhibitory effects of SGEs on DC function during collagen-induced arthritis [20]. Furthermore, both ADO and an A2_A_R agonist enhanced PGE_2_ and IL-10 production by LPS-stimulated BMDCs [20]. Therefore, it seems likely that A2_A_R is responsible for the effects of ADO on DCs. Likewise, it was recently demonstrated that an ADO A2_A_R agonist attenuated acute kidney injury by inducing tDCs [56].

In conclusion, the results presented here indicate that ADO and AMP—which are present in *P*. *papatasi* SGEs—mediate the immunosuppressive effects of saliva during leishmaniasis. ADO and AMP act through A2_A_R to induce a tDC profile by sequential production of PGE_2_ and IL-10. Both mediators could also act in a paracrine manner to induce Tregs from Teff populations, thus leading to suppression of the immune response. Understanding the molecular mechanisms induced by salivary components such as ADO and AMP—which lead to suppression of effector responses against pathogens—will help not only to understand disease pathogenesis but also to develop new vaccine strategies for cutaneous leishmaniasis.

## Supporting Information

S1 Fig Strategy gate for identification of inflammatory leucocytes during *Leishmania amazonensis* infection. For the leukocyte identification, the inflammatory cells were firstly gated based on their characteristic size (FSC) and granularity (SSC) (panel A). As gate strategy for analyzing Treg, CD3^+^ cells were gated on G1 (lymphocyte gate) (panel A) and CD4^+^CD25 subsets were determined on G2 gate (panel B). Afterwards, the Tregs markers were analyzed on CD4^+^CD25^+^ gate (G3) or under CD4^+^CD25^-^ gate (G4) (panel C). For dendritic cells analyses, the CD11c^+^ cells were gated on G5 (panel A) and subsequent activation markers (CD11c^high^MHC-II^+^) were identified individually under G6 (panel D).
(TIF)
