# Supplementary figures and images for: Nucleosides Present on Phlebotomine Saliva Induce Immunossuppression and Promote the Infection Establishment
Source: PLoS Negl Trop Dis. 2015 Apr 7;9(4):e0003600. doi: 10.1371/journal.pntd.0003600 (PMC4388340; doi:10.1371/journal.pntd.0003600)

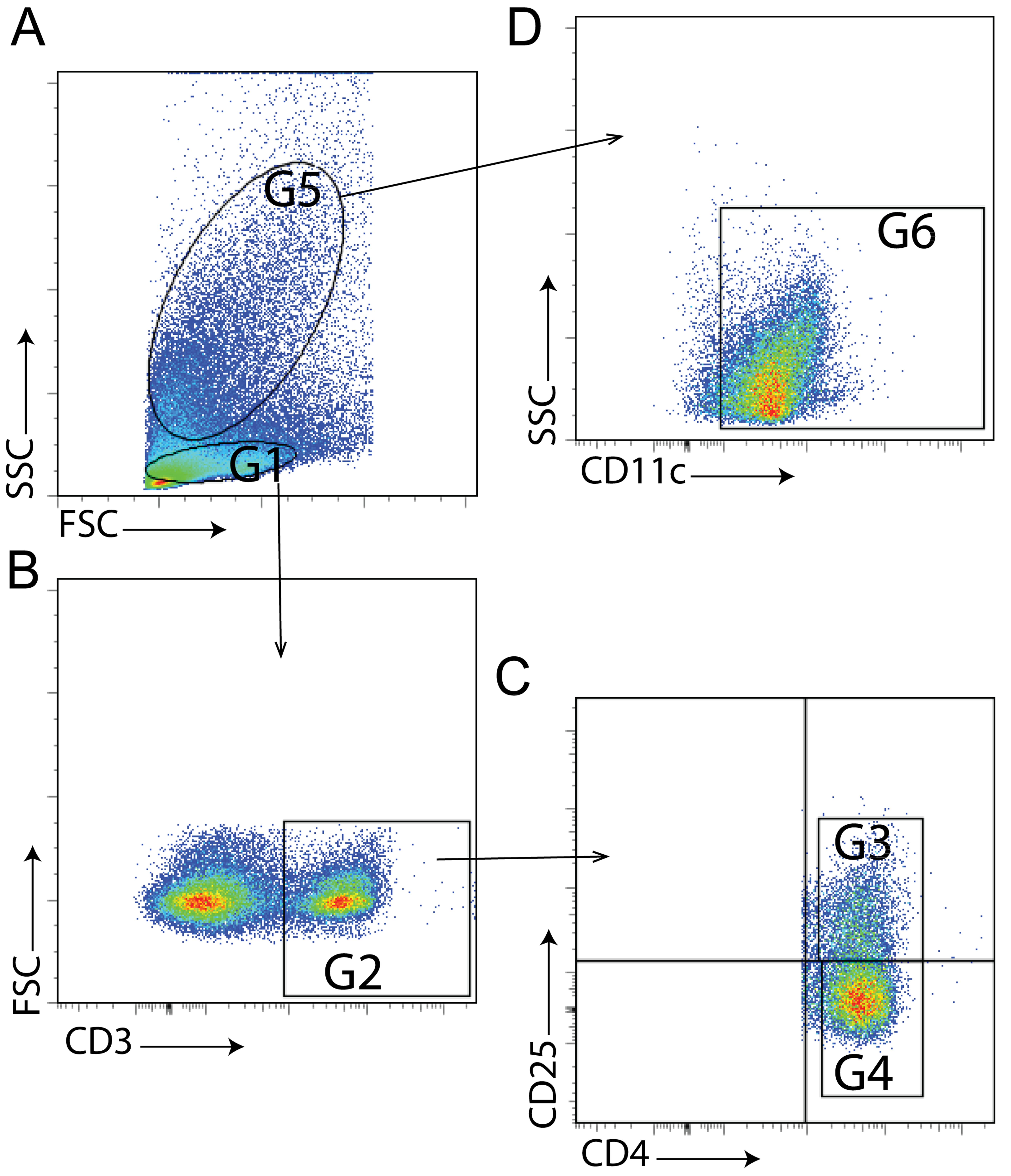

Supplement: S1 Fig — For the leukocyte identification, the inflammatory cells were firstly gated based on their characteristic size (FSC) and granularity (SSC) (panel A). As gate strategy for analyzing Treg, CD3+ cells were gated on G1 (lymphocyte gate) (panel A) and CD4+CD25 subsets were determined on G2 gate (panel B). Afterwards, the Tregs markers were analyzed on CD4+CD25+ gate (G3) or under CD4+CD25- gate (G4) (panel C). For dendritic cells analyses, the CD11c+ cells were gated on G5 (panel A) and subsequent activation markers (CD11chighMHC-II+) were identified individually under G6 (panel D). (TIF) [file pntd.0003600.s001.tif]
